# Viewpoint: The impending pandemic of resistant organisms – a paradigm shift towards source control is needed

**DOI:** 10.1097/MD.0000000000039200

**Published:** 2024-08-02

**Authors:** Kevin T. Kavanagh, Matthias Maiwald, Lindsay E. Cormier

**Affiliations:** a Health Watch USA, Lexington, KY; b Department of Pathology and Laboratory Medicine, KK Women’s and Children’s Hospital, Singapore; c Department of Microbiology and Immunology, National University of Singapore; and Duke-National University of Singapore Graduate Medical School, Singapore; d Health Watch USA, Department of Pharmacology and Nutritional Sciences, University of Kentucky, Lexington, KY.

**Keywords:** ADI, bathing, Candida auris, CDC, chlorhexidine, contact precautions, EBP, enhanced barrier precautions, HICPAC, isolation, MRSA, MSSA, NHS, surveillance, VHA

## Abstract

The United States needs a paradigm shift in its approach to control infectious diseases. Current recommendations are often made in a siloed feedback loop. This may be the driver for such actions as the abandonment of contact precautions in some settings, the allowance of nursing home residents who are carriers of known pathogens to mingle with others in their facility, and the determination of an intervention’s feasibility based upon budgetary rather than health considerations for patients and staff. Data from both the U.S. Veterans Health Administration and the U.K.’s National Health Service support the importance of carrier identification and source control. Both organizations observed marked decreases in methicillin-resistant *Staphylococcus aureus* (MRSA), but not methicillin-susceptible *Staphylococcus aureus* infections with the implementation of MRSA admission screening measures. Facilities are becoming over-reliant on horizontal prevention strategies, such as hand hygiene and chlorhexidine bathing. Hand hygiene is an essential practice, but the goal should be to minimize the risk of workers’ hands becoming contaminated with defined pathogens, and there are conflicting data on the efficacy of chlorhexidine bathing in non-ICU settings. Preemptive identification of dedicated pathogens and effective source control are needed. We propose that the Centers for Disease Control and Prevention should gather and publicly report the community incidence of dedicated pathogens. This will enable proactive rather than reactive strategies. In the future, determination of a patient’s microbiome may become standard, but until then we propose that we should have knowledge of the main pathogens that they are carrying.

**Bullet Points**

1. Strategies should have input from a variety of experts, workers and clinicians.
2. Randomized controlled trial biases may decrease the validity of their findings.
3. Hand hygiene is important, but so is carrier identification.
4. Infections and carriage of pathogens should not be normalized.
5. Cost effectiveness should be measured in terms of patient lives and livelihood.

## 1. Introduction

The United States may be on the verge of losing important ground in the fight against infectious diseases.^[1]^ The U.S. is currently battling an expanding epidemic of drug-resistant organisms and an ongoing SARS-CoV-2 pandemic. This commentary puts forward the case that many facilities have implemented ineffective strategies that are based upon research and recommendations that are not sufficiently solid and conflict with the reality that staff and patients are facing. The process of recommendation-making does not appear to be based on a wide range of expertise and stakeholders, but instead appears to be based upon the achievement of short-term goals and economic profitably. A recent commentary in the British Medical Journal regarding U.S. workers stated, “Some employers, with the support (and encouragement) of elected officials, put production and profits ahead of worker safety and health.”^[2]^ In the healthcare sector, concerns regarding the placement of profits ahead of healthcare quality have increased with the purchase of healthcare systems by private equity firms, and associated increases in adverse events, such as falls and central line infections.^[3]^

Recommendations and warnings are sometimes in conflict. For example, the Centers for Disease Control and Prevention (CDC) is currently advising that nursing homes can adopt enhanced barrier precautions,^[4,5]^ which would allow patients who are colonized with known pathogens such as *Candida auris* to take part in normal activities. However, at the same time the CDC is alerting the public to the risk of outbreaks of *Candida auris*.^[6,7]^ An alternative approach would be to attempt decolonization of carriers, and if this fails, to cohort carriers with residents who have a compatible microbiome.

For the purpose of this commentary, we use the term “Source Control” in an epidemiological context. As such, source control aims to limit the spread of known and defined pathogens to non-colonized or uninfected people. A well-known example is the Dutch “Search and Destroy” policy.^[8]^ Our aim is to put forward the case that source control is of utmost importance, and applying the CDC-defined enhanced barrier precautions appears to be counterproductive to achieving this. Recently, the CDC asked their Healthcare Infection Control Practices Advisory Committee (HICPAC) if source control should have broader recommendations and if it should be implemented at all times in healthcare facilities.^[9]^ This is an important first step, but a number of barriers still need to be overcome:

## 2. Siloed feedback loop

Some of the approval and implementation of suboptimal strategies may have its roots in the narrowing of expertise in advisory panels, such that most panel members share common opinions and biases. This sets the stage for reinforcing the validity of proposed strategies, and advancing strategies which are based upon a narrow review of scientific evidence.

Concerns that this process was taking place in the CDC’s HICPAC committee was expressed in a petition of nearly 11,000 individuals, 11 labor unions and 45 public health, patient/worker advocacy organizations, representing over 6 million members.^[10]^ There were concerns regarding the HICPAC’s recent recommendation for approval of draft guidelines for isolation precautions and the composition of the advisory committee.^[11]^

HICPAC’s recommendations appeared to be inconsistent with accepted aerosol science in terms of preventing the spread of airborne diseases, along with a homogenous membership of the Committee, which was primarily represented by healthcare industry. The HICPAC recommendations were passed unanimously, but were then sent back by the CDC leadership for reconsideration.^[9]^ Major concerns and questions HICPAC was tasked to address included:

- “Should N95 respirators be recommended for all pathogens that spread by the air?”^[9]^
- “Should source control be recommended at all times in healthcare facilities?”^[9]^
- And concerns that long COVID would not be considered in recommendations designed to prevent a severe illness.

In an apparent recognition of the effects of siloed decision-making, the CDC leadership also stated it was:

“…*working to expand the scope of technical backgrounds of participants on the HICPAC Isolation Guideline Workgroup and eventually among the committee members through established processes in accordance with the Federal Advisory Committee Act (FACA) regulations and guidance*.”^[9]^

Expanding the technical backgrounds of advisory committee members to include, for example, healthcare workers, industrial hygienists, patient safety advocates, occupational medicine experts and aerosol scientists, should bring new viewpoints and expertise to the table,^[10]^ which will help broaden the available viewpoints and facilitate the formulation of more effective strategies.

## 3. Reliance upon randomized controlled trials in public health

The United States’ response to infectious diseases has also been adversely affected by the common insistence on the availability of actionable data from randomized controlled trials (RCTs). However, RCTs may be inadequately suited for the validation of public health interventions. They may not be feasible, either when they would require very large numbers of participants to obtain meaningful results, or when there is agreement in the healthcare community that the control group would have a higher a priori risk of adverse outcomes than the intervention group.^[12]^ RCTs also often contain biases which cannot be controlled for, or ethically eliminated. There is also a risk that the absence of supportive RCTs may be used as an excuse to not implement more costly but nevertheless effective control strategies.

For example, recent practice recommendations to designate contact precautions as “essential” for the prevention of Methicillin-resistant Staphylococcus aureus (MRSA) transmission and infection specified exceptions for certain healthcare facilities.^[13]^ A commentary published in Clinical Infectious Diseases went further and questioned the essential nature of contact precautions, arguing that they should be considered “additional approaches.”^[14]^ However, 2 out of several cited RCTs (e.g. STAR*ICU, REDUCE-MRSA, & MOSAR studies) may raise concerns.

The STAR*ICU study appeared to not have effectively initiated or implemented contact precautions.^[15]^ The study’s lead author stated in an amednews.com interview^[16]^ that:

*“All nasal swabs were sent to the National Institutes of Health’s Clinical Microbiology Laboratory for culture testing, creating a five-day wait that delayed implementation of the added infection-control measures. Also, adherence to the hygiene standards was less than perfect. Health professionals wore gloves 82% of the time they interacted with colonized or infected patients, donned gowns 77% of the time and cleaned their hands 69% of the time after leaving patient rooms.”*

In the REDUCE-MRSA study,^[17]^ the reduction in “all-cause” bloodstream infections in the universally chlorhexidine-bathed trial arm referred to an aggregate category which was added after the trial completion date.^[18,19]^ Yeast species and commensal bacteria appeared to be contributing most to the significant decrease in “all-cause” bloodstream infections. A significant decrease was not specifically observed for MRSA bloodstream infections. In this regard, this was a negative study.

The MOSAR study^[20]^ found no additional decrease in MRSA acquisitions associated with screening-guided contact precautions, compared to the initial reduction achieved by the hand hygiene and chlorhexidine bathing intervention. The patients studied were critically ill ICU patients, with 60% having endotracheal tubes and 70% central lines.^[21,22]^ Care bundles and best practices were carried out in both arms, which may have mitigated the added benefit of contact precautions, and extrapolation of the results to other hospital and nursing home settings needs to be done with caution.

In contrast, the Northwestern-MRSA^[23]^ and the Leonhardt et al^[24]^ clinical trials observed a beneficial effect with surveillance followed by contact precautions. However, Leonhardt et al^[24]^ compared targeted versus universal surveillance, and this did not reach statistical significance. In the Agency for Healthcare Research and Quality’s (AHRQ’s) analysis of 42 studies,^[25]^ only the Swiss-MRSA^[26]^ and STAR*ICU^[15]^ studies did not show a beneficial effect of surveillance. However, both studies appeared to have biases. The majority of patients in the Swiss-MRSA^[26]^ study’s intervention group did not receive antibiotics effective against MRSA. Of the remaining 40 studies reported by AHRQ, many were before and after studies, but all showed a positive result, with 30 reporting statistical significance. It is unlikely that not controlling secular trends allowed random biases to be present, since all would have to be in the same direction.

During the time period of the Swiss-MRSA and Star*ICU Studies, it was “strongly recommended” by the CDC that acute care facilities “Implement Contact Precautions routinely for all patients infected with target MDROs and for patients that have been previously identified as being colonized with target MDROs,” including MRSA.^[27,28]^

Another recent example of a RCT that may have contained a bias was an article by Miller et al, evaluating chlorhexidine bathing and decolonization of nursing home residents.^[29]^ The intervention arm of the study received “...in-person training sessions, coaching calls, and a toolkit of protocols, training materials and staff and resident handouts” but the control arm did not receive such supporting measures. The authors stated that their findings were supported by those of the ABATE infection trial,^[30]^ which observed a reduction of multidrug-resistant organism (MDRO) positive clinical cultures and all-pathogen bloodstream infections in patients with medical devices. However, the positive finding in the ABATE study was a post hoc analysis of the data, and the ABATE study was not able to achieve its primary outcome of a reduction of MRSA or vancomycin-resistant enterococci clinical cultures in the general hospital population, nor did it achieve the secondary outcome of reduction of all-pathogen bloodstream infections in that population. The authors postulated that the negative results might have arisen because in the control group they “allow[ed] and expect[ed] hospitals to organize campaigns to improve adherence to existing best practices.” One might argue that if the promotion of existing best practices in the control arm was responsible for the negative results in the ABATE study, that the absence of this promotion in the control arm of the Miller et al study could be a reason for this study’s positive results.

Combined with the lack of significant MRSA bloodstream infection prevention as reported in the Reduce MRSA study,^[17]^ there are questions whether chlorhexidine bathing is a viable strategy to control the spread of major pathogens in non-ICU settings.

## 4. Hand hygiene is not a substitute for surveillance with isolation or decolonization

Hand hygiene is an essential preventative measure; it is part of standard precautions that should be applied in the care of all patients. However, it has known gaps in compliance and our goal should be to minimize the risk of healthcare workers’ hands to become contaminated with defined pathogens. Hand hygiene should therefore be viewed as complementary to, not a substitute for, surveillance strategies. Hand hygiene is ineffective in stopping the spread of airborne pathogens which can be spread by shouting, singing, talking, and breathing. In addition, aerosolizing procedures may aerosolize almost all pathogens.

One of the strongest known tools for source control is surveillance and the identification of carriers. This is important in preventing outbreaks of both viral and bacterial pathogens.

The importance of surveillance is illustrated by the difference in the rates of MRSA infections in the Veterans Health Administration (VHA) compared to the private sector. From 2005 to 2017, the VHA saw a continued decrease in MRSA hospital-onset infections associated with the implementation of universal screening of hospital admissions and the implementation of contact precautions for identified carriers.^[31]^ In private facilities, MRSA bloodstream infection rates had a modest decrease of less than 20%,^[32]^ and then during the COVID-19 pandemic increased to approximately 17% above their 2010 to 2011 baseline levels.^[32]^ However, the VHA maintained a level which was more than 80% below their baseline.^[32]^

In the Clinical Infectious Diseases’ commentary,^[14]^ the explanation was advanced that the VHA’s decrease in MRSA infections may not have been due to contact precautions, but to an increase emphasis the VHA placed on “horizontal measures,” such as hand hygiene during this time period. However, if the decrease in MRSA was due to horizontal interventions, then a commensurate decrease in Methicillin-susceptible Staphylococcus aureus(MSSA) should have been seen. This was not the case. In reporting the VHA data, Jones et al^[33]^ stated that the implementation of multi-faceted control interventions:

*“...included admission screening for nasal MRSA carriage and use of contact precautions for MRSA-colonized patients, VAMCs across the United States experienced a sharp decline in S. aureus infections among hospitalized patients. Most of the reductions were explained by decreases in MRSA; reductions in MSSA rates were more modest.”*

Similar results have been reported in the United Kingdom during the implementation of admission screening for MRSA.^[34]^ MRSA bloodstream infections dropped approximately 75%, but there was “no reduction” seen with MSSA infections.

## 5. The normalization of deviance, lessons learned from MRSA

The United States’ apparent lack of ability to control the high rates of drug-resistant infections occurs hand in hand with an apparent willingness to accept the status quo. It appears that some infectious disease authorities have become complacent and may express acceptance of known pathogens residing on healthcare workers’ hands and work attire. However, not just humans but also facilities have a resident microbiome, and the goal should be for this to be composed of mainly commensal organisms. Contamination with defined pathogens, such as MRSA, CRE, or SARS-CoV-2 should be minimized to the greatest extent possible. Otherwise, the World Health Organization’s goal of “preventing all infections” becomes harder to achieve.

We find it not acceptable to be advocating that facility interventions should only be triggered by disease (e.g. MRSA) outbreaks. This assertion has been used by some authors in the context of when to initiate MRSA surveillance and contact precautions. This approach is not only reactive, but when to initiate interventions is unclear, since there is not an accepted definition for an outbreak. The definition of an outbreak is often defined by the facility.

We think it is unlikely that the United States will be able to drive down rates of MDRO infections by reactive strategies. Knowledge of colonization rates in the community appears key to initiating proactive strategies. When an outbreak occurs in a facility, this means there is already a failure. Healthcare authorities, including the CDC, do not appear to be in possession of community prevalence or carrier rates for many defined pathogens, including MRSA.

Reactive strategies also appear to have been advocated for contact precautions for MRSA.^[13]^ A recent statement by SHEA/IDSA and APIC states:

*“Although contact precautions remain an essential practice, considerations have been provided for hospitals that have strong horizontal prevention measures and neither ongoing MRSA outbreaks nor high or increasing rates of MRSA infection or hospital-onset MRSA-positive cultures and that choose to modify the use of contact precautions for some or all MRSA-colonized or MRSA-infected patients.”*

The Clinical Infectious Diseases commentary^[14]^ interpreted this in the following way: *“…the compendium authors themselves concede that CP [contact precautions] for all patients with MRSA may not be ‘essential’ by devoting considerable attention to hospitals that have (or will) move on from routine use of CP for MRSA.”*

However, one needs to ask, for the healthcare worker taking care of a patient with an MRSA infection, what difference does the number of other MRSA patients in the hospital make for the precautions that workers will take in treating his or her patients? MRSA is contagious and acquisition by healthcare workers and others in the facility carries substantial risks.^[35,36]^

Another argument for not controlling the spread of MRSA is that it is usually not more lethal than MSSA. Although the validity of this statement may depend upon the strain of MRSA, both MRSA and MSSA can cause serious disease and death. This argument ignores the risk of allowing MRSA to spread and evolve in a hospital setting, which may result in more highly resistant strains, such as vancomycin-resistant *Staphylococcus aureus* (VRSA), becoming prevalent.

## 6. Cost effectiveness (Burden) of interventions

Budgetary considerations appear too often to be based on safeguarding the profits of corporate owners and stockholders, along with the salaries of healthcare system administrators.^[3]^ A congressional bipartisan probe is investigating this concern. As recently stated by U.S. Senator Chuck Grassley “When it comes to our nation’s hospitals, a business model that prioritizes profits over patient care and safety is unacceptable.”^[37]^

Being too burdensome is often used to justify not implementing certain strategies. Cost effectiveness has almost always been calculated from the point of view of the institution, and whether the proposed intervention costs less to deliver than usual care. However, when viewed in terms of burden to society, what is burdensome takes on an entirely new perspective. The United States’ Department of Health and Human Services has determined that for every life lost, society loses 9.6 million dollars.^[38]^ And every year of disability (loss of a quality life year) would equate to almost 500,000 dollars.

The fallacy of our current view of “burdensome” is apparent when this is applied to the prevention of SARS-CoV-2 infections in healthcare settings. The effects of long COVID are adversely impacting our workforce. One hospital system in Germany reports that 50% of its staff has been affected by long COVID as defined as post-COVID symptoms lasting at least 3 months.^[39]^ Almost all had been affected with “mild acute COVID-19.” It would be expected that over 40% of those afflicted will have symptoms after a year.^[40,41]^ Unfortunately, no one is truly tracking healthcare worker acquisitions of COVID-19 in our workforces, and only a small fraction of patient acquisitions are reported. During the pandemic, only currently hospitalized patients who developed COVID-19 14 days or more after admission were reported.^[42]^ In June of 2023, reporting of hospital-acquired COVID-19 became no longer mandatory.^[43]^

In the short term, an intervention may be viewed as not being “cost effective,” but if the lack of implementation has a negative effect on the healthcare workforce and our society, the intervention will become “cost effective” over the median and long term.

## 7. Conclusions

Source control is of utmost importance. One might argue that knowledge of the composition of every patient’s microbiome is unlikely to be available in the near future. However, we should at least be testing for major pathogens which are found in the community. This would include both MRSA and MSSA, along with some common viral pathogens. We argue that we must not accept the status quo, but endeavor to drive down the current rates of infections. Horizontal measures should complement and not be a substitute for targeted preventive interventions. A multifaceted strategic approach is needed. The healthcare system needs a paradigm shift in how it views what is burdensome, and additional strategies need to be implemented to mitigate our current pandemic of drug-resistant bacterial and viral infections, allowing us to preserve a thriving society.

## Author contributions

**Conceptualization:** Kevin T. Kavanagh.

**Data curation:** Kevin T. Kavanagh, Matthias Maiwald.

**Resources:** Lindsay E. Cormier.

**Software:** Lindsay E. Cormier.

**Writing – original draft:** Kevin T. Kavanagh.

**Writing – review & editing:** Kevin T. Kavanagh, Matthias Maiwald, Lindsay E. Cormier.

## References

[1] World Health Organization. Antibiotic resistance. World Health Organization; 2023. [updated Nov. 21, 2023]. https://www.who.int/news-room/fact-sheets/detail/antimicrobial-resistance. Accessed May 10, 2024.

[2] Michaels D Spieler EA Wagner GR. US workers during the covid-19 pandemic: uneven risks, inadequate protections, and predictable consequences. BMJ. 2024;384:e076623. 38286467 10.1136/bmj-2023-076623

[3] Kannan S Bruch JD Song Z. Changes in hospital adverse events and patient outcomes associated with private equity acquisition. JAMA. 2023;330:2365–75. 38147093 10.1001/jama.2023.23147 PMC10751598

[4] Centers for Disease Control and Prevention. Consideration for use of enhanced barrier precautions in skilled nursing facilities. Centers for Disease Control and Prevention; 2021. https://www.cdc.gov/hicpac/media/pdfs/EnhancedBarrierPrecautions-508.pdf. Accessed July 23, 2024.

[5] Centers for Disease Control and Prevention. Frequently Asked Questions (FAQs) about enhanced barrier precautions in nursing homes. Centers for Disease Control and Prevention; 2022. [updated July 27, 2024]. https://www.cdc.gov/long-term-care-facilities/hcp/prevent-mdro/faqs.html. Accessed July 23, 2024.

[6] Centers for Disease Control and Prevention. Antimicrobial Resistance Threats in the United States, 2021-2022. Centers for Disease Control and Prevention; 2024. https://www.cdc.gov/antimicrobial-resistance/media/pdfs/antimicrobial-resistance-threats-update-2022-508.pdf. Accessed July 23, 2024.

[7] Centers for Disease Control and Prevention. Increasing threat of spread of antimicrobial-resistant fungus in healthcare facilities [press release]. Centers for Disease Control and Prevention; 2023. https://www.cdc.gov/media/releases/2023/p0320-cauris.html. Accessed May 10, 2024.

[8] Vos MC Behrendt MD Melles DC. 5 years of experience implementing a methicillin-resistant Staphylococcus aureus search and destroy policy at the largest university medical center in the Netherlands. Infect Control Hosp Epidemiol. 2009;30:977–84. 19712031 10.1086/605921

[9] Jernigan D Howard J. A CDC update on the draft 2024 guideline to prevent transmission of pathogens in healthcare settings. Centers for Disease Control and Prevention; 2024. January 23, 2024. https://blogs.cdc.gov/safehealthcare/draft-2024-guideline-to-prevent-transmission-of-pathogens-in-healthcare-settings/. Accessed May 10, 2024.

[10] National Nurses United. NNU delivers petition urging CDC to strengthen proposed infection control guidance; CDC cuts off public comments at advisory committee meeting. 2023. https://www.nationalnursesunited.org/press/nnu-delivers-petition-urging-the-cdc-to-strengthen-proposed-infection-control-guidance. Accessed May 10, 2024.

[11] Brosseau LM Gold D Materna B Rosen J Seminario P Thomason J. Public health experts ask CDC director to broaden input on revisions to key infection control guidelines. New Solut. 2023;33:165–73. 37621093 10.1177/10482911231195898

[12] Yusuf E Maiwald M. COVID-19, equipoise and observational studies: a reminder of forgotten issues. Infection. 2021;49:371–3. 32588335 10.1007/s15010-020-01466-9 PMC7315903

[13] Popovich KJ Aureden K Ham DC. SHEA/IDSA/APIC practice recommendation: strategies to prevent methicillin-resistant staphylococcus aureus transmission and infection in acute-care hospitals: 2022 update. Infect Control Hosp Epidemiol. 2023;44:1039–67. 37381690 10.1017/ice.2023.102 PMC10369222

[14] Diekema DJ Nori P Stevens MP Smith MW Coffey KC Morgan DJ. Are contact precautions “essential” for the prevention of healthcare-associated methicillin-resistant staphylococcus aureus? Clin Infect Dis. 2023;78:1289–94. 10.1093/cid/ciad571 37738565

[15] Huskins WC Huckabee CM O’Grady NP.; STAR*ICU Trial Investigators. Intervention to reduce transmission of resistant bacteria in intensive care. N Engl J Med. 2011;364:1407–18. 21488763 10.1056/NEJMoa1000373 PMC3410743

[16] O’Reilly KB. Doubt cast on effectiveness of universal MRSA screening. amednewscom. 2011. https://amednews.com/article/20110426/profession/304269997/8/. Accessed May 10, 2024].

[17] Huang SS Septimus E Kleinman K.; CDC Prevention Epicenters Program. Targeted versus universal decolonization to prevent ICU infection. N Engl J Med. 2013;368:2255–65. 23718152 10.1056/NEJMoa1207290 PMC10853913

[18] Kavanagh KT Calderon LE Saman DM. Viewpoint: a response to “Screening and isolation to control methicillin-resistant Staphylococcus aureus: sense, nonsense, and evidence”. Antimicrob Resist Infect Control. 2015;4:4. 25729571 10.1186/s13756-015-0044-9 PMC4345038

[19] Platt R. Cluster randomized trial of hospitals to assess impact of targeted versus universal strategies to reduce Methicillin-resistant Staphylococcus Aureus (MRSA) in Intensive Care Units (ICUs) (REDUCE-MRSA). U.S. National Library of Medicine; 2024. https://classic.clinicaltrials.gov/ct2/history/NCT00980980?B=9&A=8&C=merged#StudyPageTop. Accessed May 10, 2024.

[20] Derde LPG Cooper BS Goossens H.; MOSAR WP3 Study Team. Interventions to reduce colonisation and transmission of antimicrobial-resistant bacteria in intensive care units: an interrupted time series study and cluster randomised trial. Lancet Infect Dis. 2014;14:31–9. 24161233 10.1016/S1473-3099(13)70295-0 PMC3895323

[21] Bonten MJ Brun-Buisson C Cooper BS. Care bundles in intensive care units – authors’ reply. Lancet Infect Dis. 2014;14:372. 10.1016/S1473-3099(14)70740-6 24758996

[22] Golzari SE Mahmoodpoor A. Care bundles in intensive care units. Lancet Infect Dis. 2014;14:371–2. 10.1016/S1473-3099(14)70731-5 24758993

[23] Robicsek A Beaumont JL Paule SM. Universal surveillance for methicillin-resistant staphylococcus aureus in 3 affiliated hospitals. Ann Intern Med. 2008;148:409–18. 18347349 10.7326/0003-4819-148-6-200803180-00003

[24] Leonhardt KK Yakusheva O Phelan D. Clinical effectiveness and cost benefit of universal versus targeted methicillin-resistant staphylococcus aureus screening upon admission in hospitals. Infect Control Hosp Epidemiol. 2011;32:797–803. 21768764 10.1086/660875

[25] Glick SB Samson DJ Huang E Vats V Weber S Aronson N. Screening for Methicillin-Resistant Staphylococcus Aureus (MRSA). Agency for Healthcare Research and Quality, U.S. Department of Health and Human Services; 2013. AHRQ Publication No. 13-EHC043-EF. https://www.healthwatchusa.org/HWUSA-Initiatives/PDF-Downloads/20130620-MRSA-screening-report-130617.pdf. Accessed May 10, 2024. 23905190

[26] Harbarth S Fankhauser C Schrenzel J. Universal screening for methicillin-resistant staphylococcus aureus at hospital admission and nosocomial infection in surgical patients. JAMA. 2008;299:1149–57. 18334690 10.1001/jama.299.10.1149

[27] Siegel JD Rhinehart E Jackson M Chiarello L, HICPAC. Management of multidrug-resistant organisms in healthcare settings, 2006. Centers for Disease Control and Prevention; 2006. https://www.cdc.gov/infection-control/media/pdfs/Guideline-MDRO-H.pdf. Accessed July 23, 2024.

[28] Centers for Disease Control and Prevention. Multidrug-resistant organisms (MDRO) management. Centers for Disease Control and Prevention; 2024. https://www.cdc.gov/infection-control/hcp/mdro-management/summary-recommendations.html. Accessed July 23, 2024.

[29] Miller LG McKinnell JA Singh RD. Decolonization in nursing homes to prevent infection and hospitalization. N Engl J Med. 2023;389:1766–77. 37815935 10.1056/NEJMoa2215254 PMC13016440

[30] Huang SS Septimus E Kleinman K.; ABATE Infection trial team. Chlorhexidine versus routine bathing to prevent multidrug-resistant organisms and all-cause bloodstream infections in general medical and surgical units (ABATE Infection trial): a cluster-randomised trial. Lancet. 2019;393:1205–15. 30850112 10.1016/S0140-6736(18)32593-5 PMC6650266

[31] Centers for Disease Control and Prevention. Staph Infections Can Kill. More prevention in healthcare and communities needed. Centers for Disease Control and Prevention; 2019 [updated March 22, 2019]. https://www.cdc.gov/vitalsigns/staph/index.html. Accessed May 10, 2024.

[32] Kavanagh KT Cormier LE. Success and failures in MRSA infection control during the COVID-19 pandemic. Antimicrob Resist Infect Control. 2022;11:118. 36153597 10.1186/s13756-022-01158-z PMC9509631

[33] Jones M Jernigan JA Evans ME Roselle GA Hatfield KM Samore MH. Vital signs: trends in staphylococcus aureus infections in veterans affairs medical centers – United States, 2005–2017. MMWR Morb Mortal Wkly Rep. 2019;68:220–4. 30845116 10.15585/mmwr.mm6809e2 PMC6421970

[34] Otter J. The English MRSA Miracle. Reflections on Infection Prevention and Control. 2015. [cited March 12, 2023]. https://reflectionsipc.com/2015/03/03/the-english-mrsa-miracle/. Accessed May 10, 2024.

[35] Chang S Sethi AK Stiefel U Cadnum JL Donskey CJ. Occurrence of skin and environmental contamination with methicillin-resistant Staphylococcus aureus before results of polymerase chain reaction at hospital admission become available. Infect Control Hosp Epidemiol. 2010;31:607–12. 20397963 10.1086/652775

[36] Neely AN Maley MP. Survival of enterococci and staphylococci on hospital fabrics and plastic. J Clin Microbiol. 2000;38:724–6. 10655374 10.1128/jcm.38.2.724-726.2000 PMC86187

[37] Morgenson G. Senators launch bipartisan probe of private equity’s growing role in U.S. health care. NBC News. 2023 Dec. 6, 2023. https://www.nbcnews.com/politics/congress/senators-grassley-whitehouse-probe-private-equity-us-health-care-rcna128070. Accessed May 10, 2024.

[38] Guidelines for regulatory impact analysis. Office of the Assistant Secretary for Planning and Evaluation, U.S. Department of Health and Human Services; 2016. https://aspe.hhs.gov/sites/default/files/migrated_legacy_files/171981/HHS_RIAGuidance.pdf. Accessed May 10, 2024.

[39] Gruber R Montilva Ludewig MV Wessels C. Long-term symptoms after SARS-CoV-2 infection in a cohort of hospital employees: duration and predictive factors. BMC Infect Dis. 2024;24:119. 38262969 10.1186/s12879-023-08710-1 PMC10807182

[40] Hartung T Bahmer T Chaplinskaya-Sobol I Deckert J Endres M. Predictors of non-recovery from fatigue and cognitive deficits after COVID-19: a prospective, longitudinal, population-based study. EClinicalMedicine. 2024;69:102456. 38333368 10.1016/j.eclinm.2024.102456 PMC10847699

[41] Kuang S Earl S Clarke J Zakaria D Demers A Aziz S. Experiences of Canadians with long-term symptoms following COVID-19. Statistics Canada; 2023. https://www150.statcan.gc.ca/n1/pub/75-006-x/2023001/article/00015-eng.htm. Accessed May 10, 2024.

[42] COVID-19 guidance for hospital reporting and FAQs for hospitals, hospital laboratory, and acute care facility data reporting. 2022. https://www.healthwatchusa.org/downloads/20221215-covid-19-faqs-hospitals-hospital-laboratory-acute-care-facility-data-reporting.pdf. Accessed May 10, 2024.

[43] COVID-19 guidance for hospital reporting and FAQs for hospitals, hospital laboratory, and acute care facility data. U.S. Department of Health and Human Services; 2023. https://www.healthwatchusa.org/downloads/20230611-covid-19-faqs-hospitals-hospital-laboratory-acute-care-facility-data-reporting.pdf. Accessed May 10, 2024.

